# Comparison of Changes in Corneal Biomechanical Properties after Photorefractive Keratectomy and Small Incision Lenticule Extraction

**DOI:** 10.4274/tjo.49260

**Published:** 2016-04-05

**Authors:** Yusuf Yıldırım, Onur Ölçücü, Abdurrahman Başcı, Alper Ağca, Engin Bilge Özgürhan, Cengiz Alagöz, Ali Demircan, Ahmet Demirok

**Affiliations:** 1 Beyoğlu Eye Training and Research Hospital, Ophthalmology Clinic, İstanbul, Turkey; 2 Rize State Hospital, Ophthalmology Clinic, Rize, Turkey

**Keywords:** Photorefractive keratectomy, small incision lenticule extraction, myopia

## Abstract

**Objectives:**

To compare the postoperative biomechanical properties of the cornea after photorefractive keratectomy (PRK) and small incision lenticule extraction (SMILE) in eyes with low and moderate myopia.

**Materials and Methods:**

We retrospectively examined 42 eyes of 23 patients undergoing PRK and 42 eyes of 22 patients undergoing SMILE for the correction of low and moderate myopia. Corneal hysteresis (CH) and corneal resistance factor (CRF) were measured with an Ocular Response Analyzer before and 6 months after surgery. We also investigated the relationship between these biomechanical changes and the amount of myopic correction.

**Results:**

In the PRK group, CH was 10.4±1.3 mmHg preoperatively and significantly decreased to 8.5±1.3 mmHg postoperatively. In the SMILE group, CH was 10.9±1.7 mmHg preoperatively and decreased to 8.4±1.5 mmHg postoperatively. CRF was significantly decreased from 10.8±1.1 mmHg to 7.4±1.5 mmHg in the PRK group whereas it was decreased from 11.1±1.5 mmHg to 7.9±1.6 mmHg in the SMILE group postoperatively. There was a significant correlation between the amount of myopic correction and changes in biomechanical properties after PRK (r=-0.29, p=0.045 for CH; r=-0.07, p=0.05 for CRF) and SMILE (r=-0.25, p=0.048 for CH; r=-0.37, p=0.011 for CRF).

**Conclusion:**

Both PRK and SMILE can affect the biomechanical strength of the cornea. SMILE resulted in larger biomechanical changes than PRK.

## INTRODUCTION

Photorefractive keratectomy (PRK) has been implemented effectively and reliably for many years in the treatment of myopia.^1,2^ In the PRK procedure, the laser is applied directly to the anterior corneal stroma without creating a flap.^2^ Small incision lenticule extraction (SMILE) is a newer procedure being utilized to treat myopia.^3,4^ In the SMILE technique, myopia is corrected by creating a corneal lenticule and extracting it through a small incision, also without creating a flap.^3,4^

It is known that corneal refractive surgery affects corneal biomechanical properites.5 There are many studies demonstrating that procedures involving flaps in particular have a negative impact on corneal biomechanical properties.^6,7^

The Ocular Response Analyzer (ORA; Reichert Ophthalmic Instruments, Depew, NY, USA) is a non-invasive instrument that assesses the corneal biomechanical properties corneal hysteresis (CH) and corneal resistance factor (CRF).^8^

Basically, the ORA takes two pressure measurements: the applanation pressure during the inward flexion of the cornea (P1) and the applanation pressure as the cornea returns to normal (P2). The difference between these two pressure measurements is the CH, reflecting the viscous resistance of the cornea.9 The CRF value expresses the mean corneal mechanical resistance including viscous and elastic components, and is calculated with the formula: k1 (P1-P2)+0.3*k1*P2+k2. The k1 and k2 values are calibration constants.^9^ CH and CRF are known to decrease in glaucoma, keratoconus and after corneal refractive surgery.^10,11,12^

The aim of this study was to compare the changes in corneal biomechanical properties after PRK and SMILE in the treatment of low and moderate myopia.

## MATERIALS AND METHODS

This retrospective study was conducted in the Refractive Surgery Unit of the Beyoğlu Eye Training and Research Hospital. The study was approved by the institutional ethics board and adhered to the tenets of the Helsinki Declaration. Myopic patients with spherical values between -2.00 and 6.00 diopters (D) and astigmatism of less than 0.50 D who underwent SMILE or PRK were included in the study. Other inclusion criteria of the study were a mesopic (4 lux) pupil diameter ≤6.5 mm and a residual stromal thickness >300 µm. Patients with previous ocular surgery, concurrent ocular disease, concurrent systemic disease (diabetes mellitus, collagen tissue disease, etc.) or contraindication to refractive surgery were excluded from the study. Patients who developed intra- or postoperative complications were also excluded.

Corneal biomechanical properties were evaluated preoperatively and 6 months postoperatively. The amount of myopic correction achieved with the procedure was recorded. In addition, the maximum ablation amount in the PRK group and the maximum lenticule thickness in the SMILE group were recorded as the amount of stromal tissue removed.

Emmetropia was the aim for all patients.

Forty-two eyes of 23 patients (12 female, 11 male) in the PRK group and 42 eyes of 22 patients (12 female, 10 male) in the SMILE group were evaluated retrospectively. The mean ages of the PRK and SMILE groups were 27.6±5.2 years and 29.0±5.9 years, respectively (p=0.23). The PRK and SMILE groups had comparable amounts of refractive correction (p=0.25). The amount of stromal tissue removed was significantly greater in the SMILE group compared to the PRK group (p=0.04). The patients’ demographic and preoperative corneal characteristics are shown in Table 1.

### Surgical Procedure

All surgical procedures were performed by the same surgeons (A.A., A.D. and E.B.Ö.). The Visumax (Carl Zeiss Meditec) femtosecond laser system was used for the SMILE procedure. Spot size was 3 µm for the lamellar cut and 2 µm for the side cut; the energy level was adjusted to 140 nanojoules (nJ). The lenticule side cut was 15 µm thick with an angle of 120° and the optical zone was 6.5 mm. The side cut was 3 mm in all eyes.

The PRK procedure was performed by first marking an area of 9 mm diameter on the anterior corneal surface and debriding the epithelium with an axe blade, followed by laser application with the AMARIS excimer laser (SCHWIND eye-tech-solutions GmbH&Co. KG, Mainparkstrasse, Kleinostheim, Germany) to a 6.5 mm optical zone. In all patients, 0.02% mitomycin C (MMC) was applied for 30 seconds following laser application.

### Measurement of Biomechanical Properties

All ORA measurements were taken preoperatively and 6 months postoperatively in a specially designated room by an experienced clinician. For each patient, three measurements close in value were taken. Unreliable atypical signals were not included in the analysis. Mean CH and CRF values were used in the analysis.

### Statistical Methods

Mean, standard deviation, median, minimum-maximum, rate and frequency values were used as descriptive statistics. Distribution of the variables was analyzed with Kolmogorov-Smirnov test. The Mann-Whitney U test was used to analyze quantitative data. Spearman correlation analysis was used to assess correlations. The Wilcoxon test was used to analyze repeated measures. Analyses were conducted using Statistical Package for the Social Sciences version 22.0 software.

## RESULTS

In the PRK group, mean CH values were 10.4±1.3 mmHg (range, 8.0-14.3 mmHg) preoperatively and 8.5±1.3 mmHg (range, 5.4-12.1 mmHg) 6 months postoperatively; CH was significantly lower at postoperative 6 months (p<0.001). In the SMILE group, preoperative CH was 10.9±1.7 mmHg (range, 7.6-14.6 mmHg) and 6 months postoperative CH was 8.4±1.5 mmHg (range, 7.6-12.6 mmHg); this difference was also statistically significant (p<0.001) (Table 2).

In the PRK group, preoperative and 6 months postoperative CRF values were 10.8±1.1 mmHg (range, 8.0-13.0 mmHg) and 7.4±1.5 mmHg (range, 4.4-10.5 mmHg), respectively (p<0.001). The SMILE group had CRF values of 11.1±1.5 mmHg (7.7-14.9) preoperatively and 7.9±1.6 mmHg (5.2-11.5) at postoperative 6 months (p<0.001) (Table 3).

The pre- to postoperative changes in CH and CRF values were significantly larger in the SMILE group compared to the PRK group (CH, p=0.03; CRF, p=0.048).

Maximum ablation amount was significantly correlated with changes in corneal biomechanical properties in both the PRK and SMILE groups (PRK: CH, r=0.24, p=0.036; CRF, r=0.28, p=0.04; SMILE: CH, r=0.19, p=0.008; CRF, r=0.39, p=0.007). In both groups, the amount of correction was negatively correlated to change in CH and change in CRF (PRK: CH, r=-0.29, p=0.045; CRF, r=-0.07, p=0.05; SMILE: CH, r=-0.25, p=0.048; CRF, r=-0.37, p=0.011) (Table 4).

None of the patients exhibited iatrogenic ectasia during the 6-month postoperative follow-up period.

## DISCUSSION

The impact of corneal refractive surgeries on the biomechanical properties of the cornea has been the focus of many studies to date.^7,10,13,14,15,16,17^ Several studies have evaluated the changes in biomechanical properties resulting from laser-assisted in situ keratomileusis (LASIK) and PRK, which have been employed for many years to treat myopia, as well as the SMILE procedure, a more current treatment method.^7,10,13,14,15,16^ Although there are studies comparing LASIK with PRK and with SMILE in terms of their effects on corneal biomechanical properties,^7,16,17^ our study is the first to compare corneal biomechanical aspects of the SMILE and PRK procedures in the treatment of myopia. In the current study, CH and CRF were used to evaluate corneal biomechanical properties.

In a study by Kamiya et al.^7^ comparing PRK and LASIK, corneal biomechanical parameters (CH and CRF) were significantly lower postoperatively in both the PRK and LASIK groups, with larger decreases observed in the LASIK group. The larger effect in the LASIK group was attributed to the creation of a corneal flap. Hamilton et al.^10^ also compared PRK and LASIK and found lower CH and CRF values postoperatively, though there was no significant difference between the two procedures. Consistent with these studies, in the current study the PRK group had significantly lower CH and CRF values.

In the current study, MMC was applied postoperatively in all patients in the PRK group. It has been demonstrated that MMC application during the PRK procedure does not cause additional changes in biomechanical properties.^13,14^ In a study by Wang et al.^15^ comparing SMILE and LASIK, CH values were significantly lower after SMILE. They found that the difference in CH was especially large when correcting myopia of -6.00 D or more. The current study included patients with myopia between -2.00 and -6.00 D. Similarly, Wu et al.^16^ compared SMILE and LASIK and found reduced CH following both procedures. Agca et al.^17^ observed negative effects of both SMILE and LASIK on corneal biomechanical properties, but did not find any differences between groups in the reduction of CH and CRF. Consistent with the literature, in the current study we found significantly lower CH and CRF values in the SMILE group.

Studies have demonstrated that in LASIK and PRK, the amount of refractive error corrected is related to the changes in corneal biomechanical properties.^7,15^ In the current study we also found significant correlations between amount of refractive correction and values for CH and CRF in both groups.

Unlike other studies, in the current study the amount of stromal tissue removed was quantified as the maximum lenticular thickness in the SMILE group and as the maximum ablation depth in the PRK group, and correlation analysis was performed using these values. In both groups, the amount of tissue removed from the stroma correlated with CH and CRF values.

In the SMILE procedure, the intracorneal lenticule is removed through a small side cut (2-3.5 mm). Because no flap is created, the SMILE procedure is considered more advantageous than LASIK in terms of the conservation of corneal biomechanical stability.^16^ PRK is also used to correct myopia without the creation of a flap. Despite both procedures being ‘flap-less’, in our study we observed larger changes in the corneal biomechanical properties of the SMILE group.

In the current study, larger changes in CH and CRF were observed in the SMILE group compared to the PRK group. Studies have demonstrated that the biomechanical resistance of the cornea is greatest in its anterior third because the collagen fibrils there are denser and more tightly linked.^18,19^ In the current study, the amount of refractive correction was comparable in the PRK and SMILE groups, whereas the amount of stromal tissue removed was significantly greater in the SMILE group (p=0.04). Therefore, the larger decreases in CH and CRF we observed in the SMILE group may be related to the presence of lamellar cuts in the anterior stroma and the greater amount of stromal tissue removed in the SMILE group compared to the PRK group.

The larger changes found in the SMILE group may be due to the fact that the method involves the removal of a piece of tissue from the stroma; even without creating a flap, making a cut within the stroma disrupts the linkage of collagen fibers. This is supported by several studies comparing SMILE and flapped corneal refractive procedures in which no significant differences were detected between the changes in corneal biomechanical properties of the two groups.^15,17^

The limitations of this study are that it was not designed prospectively and did not include a comparison with a LASIK group.

## CONCLUSION

In summary, our study demonstrates that the PRK and SMILE procedures result in reduced corneal biomechanical strength in low and moderate myopia patients. With both procedures, this effect is associated with the amount of stromal tissue removed and the amount of refractive error correction.

## Ethics

Ethics Committee Approval: The study was approved by the institutional ethics board, Informed Consent: It was taken.

Peer-review: Externally peer-reviewed.

## Figures and Tables

**Table 1:**
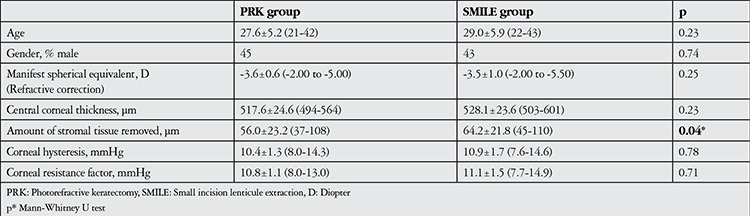
Comparison of demographic characteristics, preoperative corneal characteristics and amount of tissue removed during the procedure between the patient groups

**Table 2:**
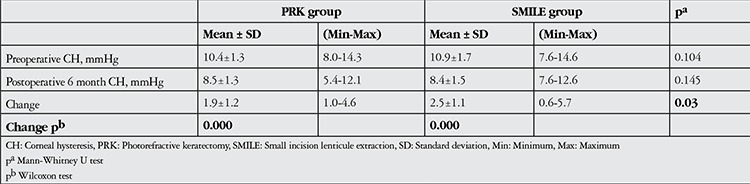
Changes in corneal hysteresis

**Table 3:**
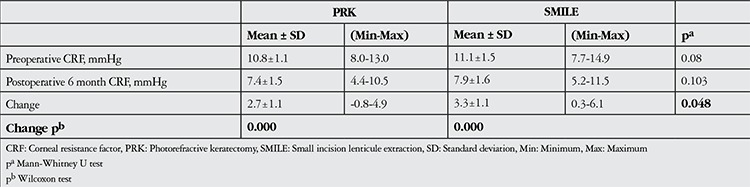
Changes in corneal resistance factor

**Table 4:**
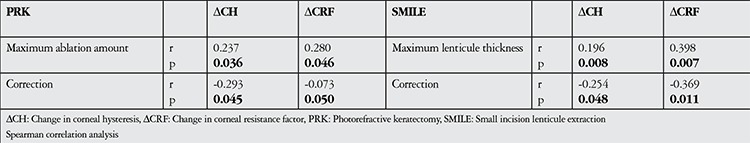
Associations between pre- to postoperative changes in corneal hysteresis and corneal resistance factor and amounts of refractive correction and stromal tissue removed
